# A Novel Feature Extraction and Fault Detection Technique for the Intelligent Fault Identification of Water Pump Bearings

**DOI:** 10.3390/s21124225

**Published:** 2021-06-20

**Authors:** Muhammad Irfan, Abdullah Saeed Alwadie, Adam Glowacz, Muhammad Awais, Saifur Rahman, Mohammad Kamal Asif Khan, Mohammad Jalalah, Omar Alshorman, Wahyu Caesarendra

**Affiliations:** 1 Electrical Engineering Department, College of Engineering, Najran University, Najran 61441, Saudi Arabia; asalwadie@nu.edu.sa (A.S.A.); srrahman@nu.edu.sa (S.R.); msjalalah@nu.edu.sa (M.J.); omar2007_ahu@yahoo.com (O.A.); 2 Department of Automatic, Control and Robotics, AGH University of Science and Technology, 30-059 Kraków, Poland; adglow@agh.edu.pl; 3 Department of Computer Science, Edge Hill University, St Helens Road, Ormskirk L39 4QP, UK; awaism@edgehill.ac.uk; 4 Mechanical Engineering Department, College of Engineering, Najran University Saudi Arabia, Najran 61441, Saudi Arabia; mkkhan@nu.edu.sa; 5 Faculty of Integrated Technologies, Universiti Brunei Darussalam, Jalan Tungku Link, Gadong BE1410, Brunei; wahyu.caesarendra@ubd.edu.bn

**Keywords:** induction motors, stator current sensing, voltage measurement, instantaneous power measurement, vibration measurement, feature selection

## Abstract

The reliable and cost-effective condition monitoring of the bearings installed in water pumps is a real challenge in the industry. This paper presents a novel strong feature selection and extraction algorithm (SFSEA) to extract fault-related features from the instantaneous power spectrum (IPS). The three features extracted from the IPS using the SFSEA are fed to an extreme gradient boosting (XBG) classifier to reliably detect and classify the minor bearing faults. The experiments performed on a lab-scale test setup demonstrated classification accuracy up to 100%, which is better than the previously reported fault classification accuracies and indicates the effectiveness of the proposed method.

## 1. Introduction

**Figure 1:**
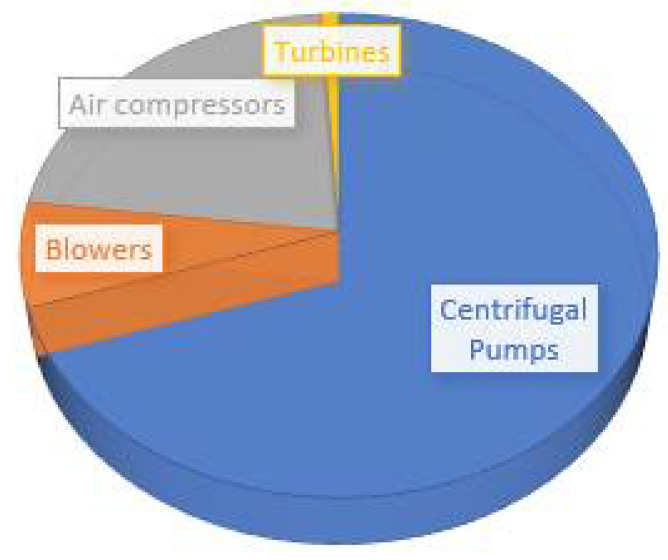
The distribution of maintenance costs in the chemical industry.

**Figure 2:**
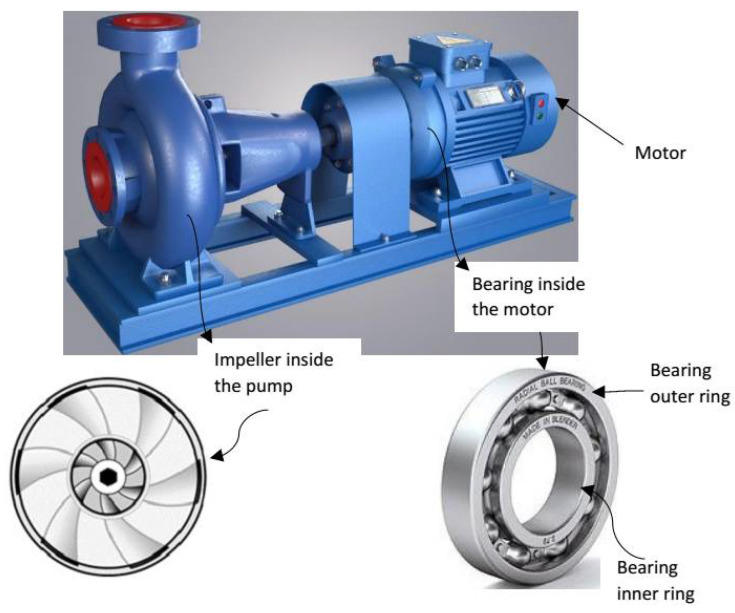
The important components of the centrifugal pump.

Induction motors have a simple, cost-effective design and they are easy to manufacture. These motors are designed to operate in tough environmental conditions. This feature makes them reliable for complex industry operations [1,2]. The stator, rotor and bearings are the main components of the induction motor. The performance of the motor is degraded if a fault appears in any of these components. Severe defects can cause a breakdown of the motor, which can create huge losses for the industry in terms of maintenance time, production stops, material waste and delay in the scheduled delivery of products [3,4,5,6,7,8]. The continuous monitoring of the status of motor parts could give an early indication about faults and provide an opportunity for the maintenance staff to assess the motor condition, take corrective measures and plan maintenance activity along with the budget. Condition monitoring of machines and systems is one of the core elements of Industry 4.0. Thanks to the digital shadow, the current status of a motor in the form of voltage, current and power or vibration could be virtually available everywhere for analysis and fault detection purposes [9,10]. Induction motors are an important component of water pumps. Condition monitoring of water pumps is very crucial to save maintenance costs and achieve the production targets of the industry. Figure 1 indicates that 70% of the maintenance cost in petrochemical plants is associated with centrifugal pumps [10]. The important elements of the centrifugal pump are shown in Figure 2. The majority of the water pumps suffer burnout due to bearing faults. The bearing faults are the cause of 44% of motor breakdowns in the industry [11] and, for this reason, the focus of this paper is on the diagnostics of bearing failures. Bearings are designed to operate for long-running hours; however, they might fail prematurely, mainly due to dirt, contamination, excessive loading, misalignment, corrosion and lack of lubrication [12]. The most common and frequent types of bearing fault reported in the literature are dents, fretting, spalling, scratches, improper fits and holes in the raceways [11,12,13,14,15].

The condition monitoring of the motor is performed through the acquisition of the data from various sensors, such as vibration sensors (accelerometer, velocity meter, displacement sensor), stator current sensors, voltage sensors, magnetic flux sensors, temperature sensors and noise sensors [16]. The name of the condition monitoring technique is decided based on the type of sensor used. Vibration analysis (VA) is a famous fault analysis technique that has been researched and practiced in the industry for a long time. The vibration information, mostly collected through accelerometers, can give information about the health of the motor [17,18]. The accuracy of the vibration analysis method is related to the accuracy of the accelerometer installation on the motor bearing. Another issue with vibration-based diagnostics is the high cost of accelerometers. Furthermore, some motors in the plant are located in places where access to the motor for accelerometer installation is nearly impossible [15,16,17,18,19,20]. Similarly, temperature analysis, noise analysis and flux analysis techniques are invasive and involve high costs for the system development [21]. The motor current analysis (MCA) technique is based on the analysis of the stator current, which is measured using current sensors, and these sensors are installed in the connection box wire terminal and thus do not require access to the motor. This feature makes MCA non-invasive and it overcomes the limitations of the VA technique [22]. The MCA supervisor decides about the health of the motor based on the amplitude of the two frequency components, which are located around the fundamental component. However, the accuracy and reliability of the fault diagnostics through MCA are affected when the amplitudes of the fault frequencies are suppressed by the high amplitude of the fundamental frequency [11]. The non-intrusive motor current analysis technique is quite inexpensive; however, its reliability is affected by environment noise while detecting minor bearing faults. Reliable fault detection and classification is a real challenge when using MCA. Researchers have achieved classification accuracy of up to 89.26% using MCA with various machine learning and deep learning tools.

The raw data obtained through sensors require further analysis to extract meaningful information about the machine’s status [23]. Various signal processing methods, such as fast Fourier transform (FFT), time-domain analysis and time-frequency analysis, have been developed and widely used in the past to analyze fault-related features such as frequency components, kurtosis, entropy and standard deviation [24,25]. The choice of the signal processing technique is depended upon the type of fault diagnosis and the nature of the fault features. 

A lot of research has been conducted in the past on the diagnosis of holes or dents in the bearing raceways and it has been shown that a small-sized hole (diameter < 1 mm) can produce tinny amplitudes; it is thus a great challenge to reliably diagnose these small faults [26,27,28,29,30]. Some studies have used noise filtration and threshold-based statistical analysis algorithms to enhance the reliability of the small-sized hole diagnostics [26,27]. Although there is a huge literature available on the diagnostics of hole-type faults in the bearing, very little research has been conducted on diagnosing the initial stage of these faults that appears in the form of scratches [28]. The unexpected breakdowns of motors in the industry could be reduced if the faults are diagnosed at the initial stage of their occurrence so that proper preventive measures can be adopted. Thus, the incipient fault diagnostics of bearings requires greater attention.

In the current decade, the use of artificial intelligence (AI) has been significantly increased for the reliable diagnostics and classification of machine faults [29]. AI techniques such as machine learning and deep learning can be trained to accomplish specific tasks by processing a large amount of data and recognizing fault trends in them [30,31,32]. There are various types of algorithms for machine learning and deep learning and the selection of the algorithm is a challenging task. Several factors, such as reliability, accuracy and processing time, should be considered to make the condition monitoring system compatible with the industry requirements. The k-nearest neighbor algorithm was used in [33] to detect and classify bearing faults and gear faults. Vibration analysis was used as a data collection technique and time-domain features were extracted and utilized for fault classification. The outer race fault size of 0.533 mm was studied; however, minor scratches diagnosis was not in the scope of this work. The performance of the k-NN algorithm was tested on various window sizes. The optimization of the model was performed through a genetic algorithm. The authors concluded that the k-NN algorithm along with a GA could be a reliable diagnostic tool for bearing condition monitoring. The support vector machine (SVM) and multilayer perception (MLP) algorithms were used in [34] to analyze centrifugal pump seal leakages. The authors used the accelerometer data collected from the installation site over four years. The SVM algorithm has been reported to achieve a maximum accuracy of 98.1% and MLP has achieved an accuracy of 98.2%. The light neural network model has been implemented on vibration signals collected from bearings and gears [35]. The authors concluded that the performance of the light neural network is better than that of other approaches. A comprehensive review of machine learning and deep learning techniques has been provided in [36]. The advantages and limitations of k-NN, naive Bayes, SVM and CNN algorithms are discussed and performance comparisons are also provided for each method.

The aim of this study was to diagnose bearing faults of minor sizes using non-invasive instantaneous power analysis (IPA) as a signal processing tool, SFSEA as a feature extraction tool and XGB as a machine learning tool. Two types of faults were simulated in the bearing: a *type 1* fault is a hole of diameter 0.5 mm and a *type 2* fault is a scratch of width 0.5 mm, depth 0.5 mm and length 5 mm. These dimensions were selected to allow a fair comparison of the proposed method with the benchmark paper [28], as this benchmark study used the same dimensions. The contributions of this paper are:

- The harmonic identification using the IPA technique. The IPA provides an advantage over conventional MCA by providing three harmonics related to fault analysis while the MCA provides only two fault-related harmonics. This extra fault harmonic helps to enhance the reliability and accuracy of the condition monitoring system.
- The design of an SFSEA for the feature extraction from the IPA data. The SFSEA extracts strong features by eliminating those features whose amplitudes are dominated by environment noise.
- The development of an XGB classifier to classify the bearing faults using the features extracted through the SFSEA.

The rest of the paper is organized as follows. Section 2 describes the SFSEA. Section 3 provides the experiment procedures and Section 4 presents the results. Finally, Section 5 presents the conclusion of this study.

## 2. Feature Selection, Extraction and Detection Framework

### 2.1. Feature Calculations and SFSEA Framework

The instantaneous power spectrum was plotted in LabVIEW through the measured voltage and current signals. The key reason to use the instantaneous power spectrum is that it gives three harmonics related to the fault. One harmonic is located at the shaft rotational frequency and two harmonics are located around the fundamental frequency. The traditional MCA technique provides only two harmonics for the fault analysis. The locations of the fault-related features in the spectrum are shown in Table 1. The mathematical relations used to calculate the locations of the fault features are described in Equations (1)–(3). In Table 1, *X*_1_ represents the harmonic at the rotational frequency and *X*_2_, *X*_3_ represent two sidebands around 100 Hz.

(1) $$Fault\;Harmonics\;\left(X_{2},\;X_{3}\right)=\left|2\times X_{f}\;\pm \;outer\;ring\;fault\;harmonic\right|$$

(2) $$Outer\;Ring\;Fault\;Harmonic\;\left(X_{1}\right)=0.4\times number\;of\;balls\times X_{r}$$

(3) $$X_{r}=\frac{X_{f}}{2}\left(1-slip\right)$$

where $X_{f}$ is the fundamental frequency and $X_{r}$ is the shaft rotational frequency.

After identifying the fault features in the instantaneous spectrum, the strong features selection and extraction algorithm was deployed in the following steps:

- The frequency spectrum is plotted using an instantaneous power algorithm developed in LabVIEW;
- The fault frequencies are identified using the mathematical model of the instantaneous power;
- The healthy bearing amplitudes are collected and saved as baseline data;
- The faulty bearing amplitudes are collected and compared with the baseline data to measure the amplitude difference (AD) (AD = measured amplitude value at characteristic frequency − baseline amplitude value at the characteristic frequency);
- If the AD is zero, then it is an indication of a healthy bearing;
- If the AD is greater than zero, then it is an indication of the presence of a fault;
- Signature with an AD is greater than zero are finally compared with the statistical threshold to eliminate the impact of the environment noise. Those signatures which are greater than the threshold are selected as the strong features and are fed to the XGB for the fault classification.

**Figure 3:**
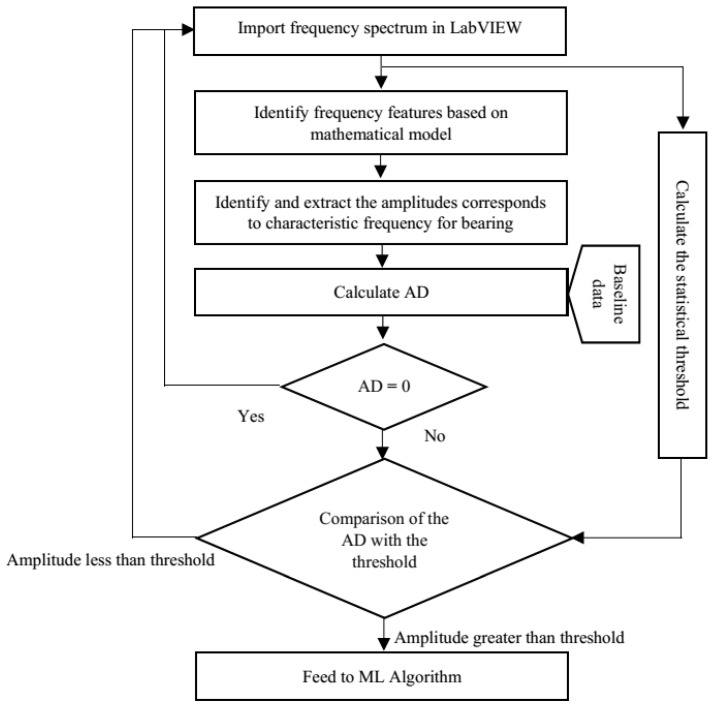
The flow chart of the SFSEA.

A flow chart of the SFSEA deployment is shown in Figure 3.

### 2.2. The Description of the Machine Learning Approaches

In general, there are two types of machine learning algorithms: supervised and unsupervised. The target variables (TVs) in supervised algorithms are those that may be predicted from a known set of independent variables. These variables are used to construct the function and input maps to get the correct result and accomplish the target. They go through a training process to improve their accuracy. The training procedure is repeated before the model reaches optimal accuracy. Unsupervised algorithms, on the other hand, do not have a TV and therefore they use the clustering strategy. The literature indicates that supervised learning is adopted for machine fault classification. The SVM, k-NN and CNN algorithms are the algorithms famous in condition monitoring applications.

#### 2.2.1. Support Vector Machine (SVM)

The SVM algorithm is primarily used for classification and regression problems. The margin and hyperplane are the fundamental parameters of the SVM algorithm. The margin identifies the dataset vectors while the hyperplane divides the datasets and executes the classification operation. The SVM algorithm classifies data by determining the best hyperplane for maximizing the margin width between various classes. The overlapping issue among various classes can be minimized by increasing the margin width. In general, there are two forms of margins: soft margins and hard margins. In the present study, a soft margin was used since the bearing fault diagnosis was a nonlinear classification problem.

Three parameters can affect the accuracy of the SVM algorithm. These parameters are the kernel function, the threshold function and the cost parameter (C). The kernel function’s primary purpose is to map the input at high-dimensional features, allowing for nonlinear classification. The gamma parameter influences data classification in the RBF kernel. The role of the threshold function is to develop the feature recognition ability. The cost parameter balances the tradeoff between a smooth boundary state determination and classification of the training points. When a large value is used as the cost parameter, low bias and high variance may be obtained. The gamma parameter sets the sequence contrast in relation to the expense function. Due to overfitting, the cost and gamma parameters should not be too large, and they should not be too poor because of underfitting issues. These parameters may be fine-tuned by choosing the right ranges of programming.

The training features are mostly used to determine the best hyperplane. Cross-validation, re-sampling and matrix scan techniques aid in the automated selection of cost and gamma parameter values during the diagnosis. In machine learning, cross-validation assists in the preparation of the algorithm utilizing the right hyper-parameters. Re-sampling is a collection of techniques for reconstructing reference data sets, such as preparation and analyzing datasets. Grid quest is an iterative approach for finding the right parameter value for a machine learning algorithm.

#### 2.2.2. K-Nearest Neighbor (k-NN) Algorithm

The k-NN algorithm is a non-parametric and robust learning algorithm that can be used to solve both classification and regression problems. Rather than studying the discriminative function, this algorithm memorizes the testing datasets. By memorizing the training sets, instance-based learning aids in the avoidance of errors. The non-parametric in the model is not set in advance and differs depending on the sample size. The drawbacks of k-NN include its huge memory footprint, long prediction period and excessive sensitivity to irrelevant functionality.

k-NN uses the k-nearest training samples around the test results to conduct classification of the data. k-NN is largely based on two factors: (1) a distance metric for measuring the distance between two points and (2) the value of “k” for defining the number of neighbors. The form of the judgment boundary is defined by the value of k. If one raises the value of k in the neighbor selection, the boundary becomes smoother. Small k values produce a hard boundary state technically, but they also result in a more stable match with low bias and large variance.

#### 2.2.3. Convolutional Neural Network (CNN) Algorithm 

The standard versions of deep learning are denoising auto-encoders, deep conviction networks and convolutional neural networks (CNNs). CNNs are often used in the medical field but seldom used for motor condition monitoring applications. CNNs are often referred to as a form of machine learning, but they belong to the AI subclass. Both controlled and unsupervised programs may be executed on the network. To put it another way, the network is made up of several layers, each of which contains secret layers that are used to train the input. A CNN was chosen from among the different architectures because of advantages such as shift-variance, weight sharing, a high accuracy rate and data encoding.

## 3. Experimental Procedure

**Figure 4:**
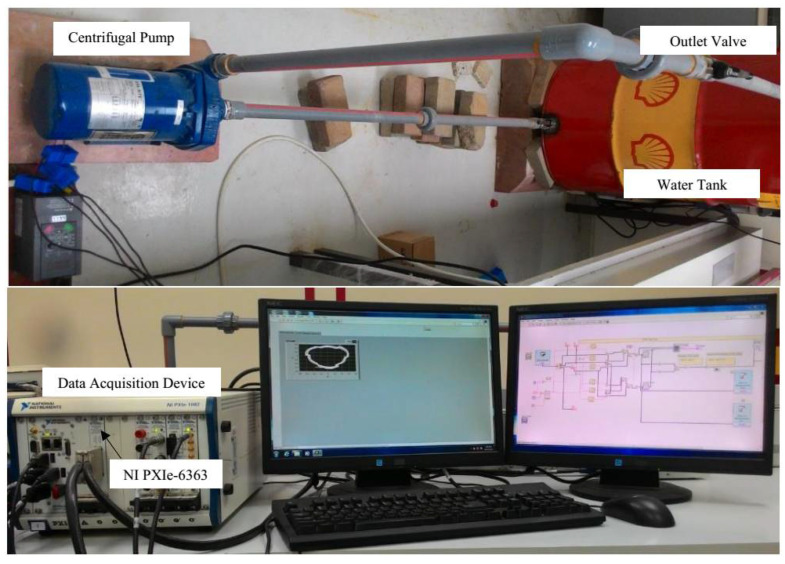
The photo of the experiment test rig.

**Figure 5:**
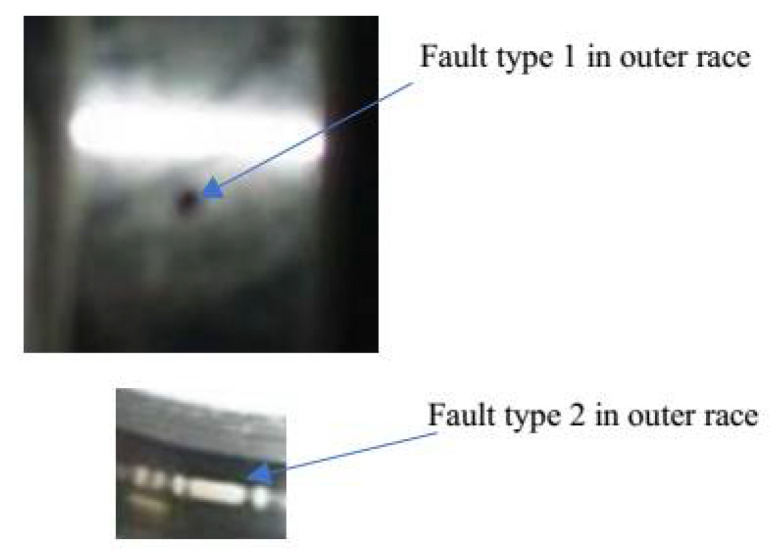
Fault types simulated in the bearing.

A current sensor (SCT-00-15) and a voltage sensor were used to collect the data from the 2 HP induction motor. The bearing used in this study was a 6202-2z bearing. It had eight balls located between the outer and inner rings. Two types of faults were simulated in the bearing: a *Type 1* fault was a hole of diameter 0.5 mm and a *Type 2* fault was a scratch of width 0.5 mm, depth 0.5 mm and length 5 mm. The data from the sensors were fed to the LabVIEW software through the National Instruments NI PXIe 1082 interface. A photo of the laboratory-scale test setup is shown in Figure 4 and simulated bearing faults are shown in Figure 5.

## 4. Results and Discussion

**Figure 6:**
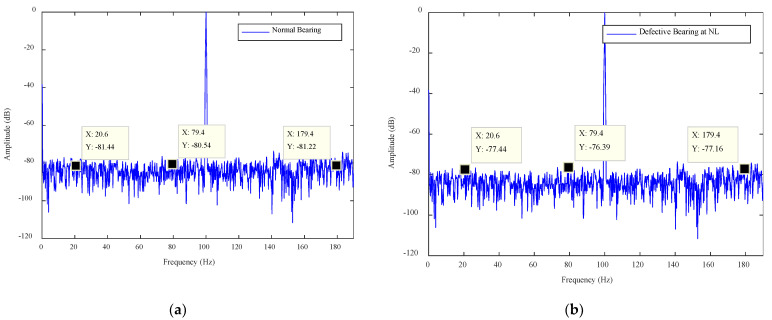

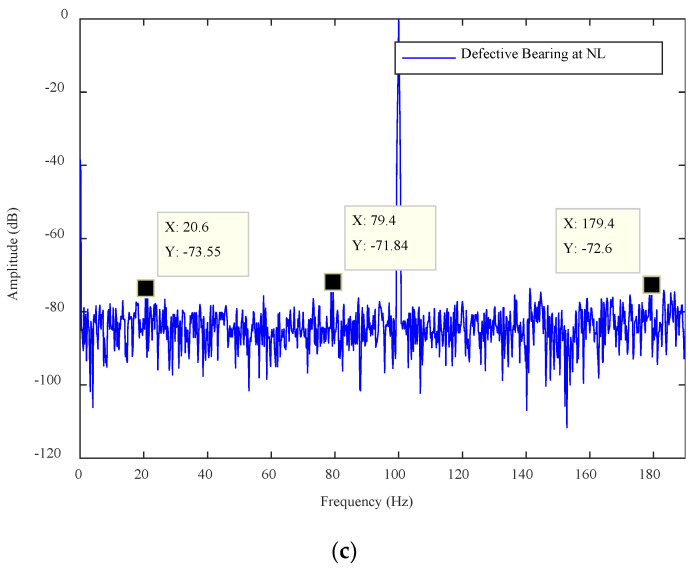
The NL plots for the (**a**) normal bearing, (**b**) type 1 defect and (**c**) type 2 defect.

**Figure 7:**
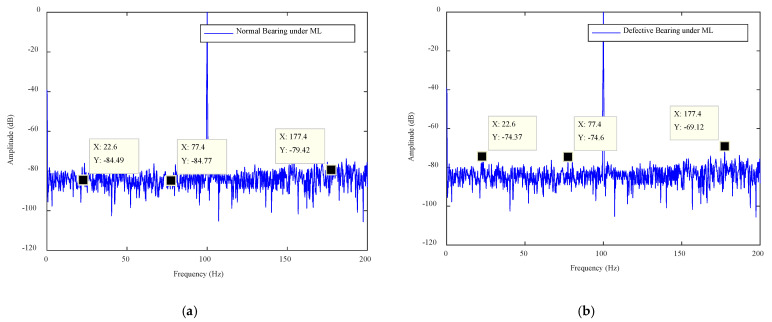

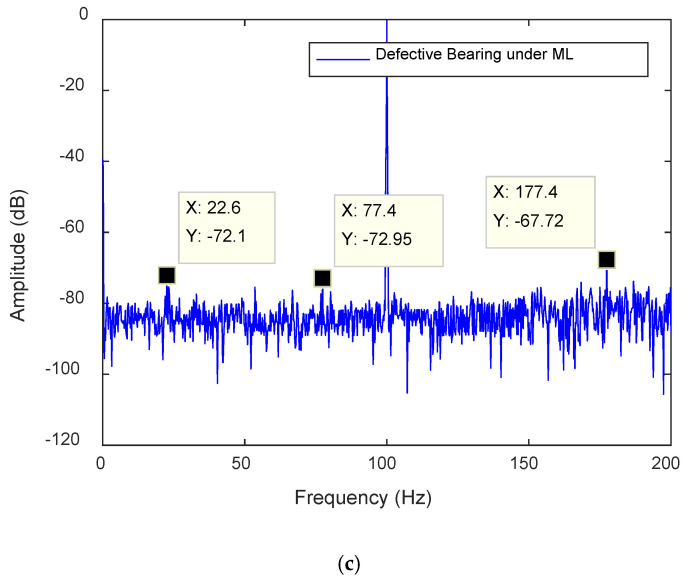
The ML plots for the defective bearing: (**a**) normal bearing, (**b**) type 1 defect and (**c**) type 2 defect.

**Figure 8:**
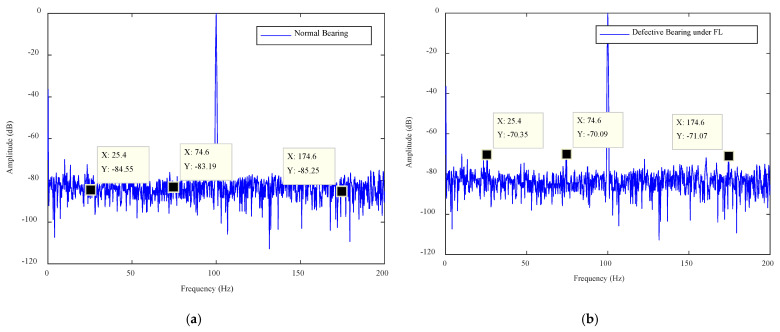

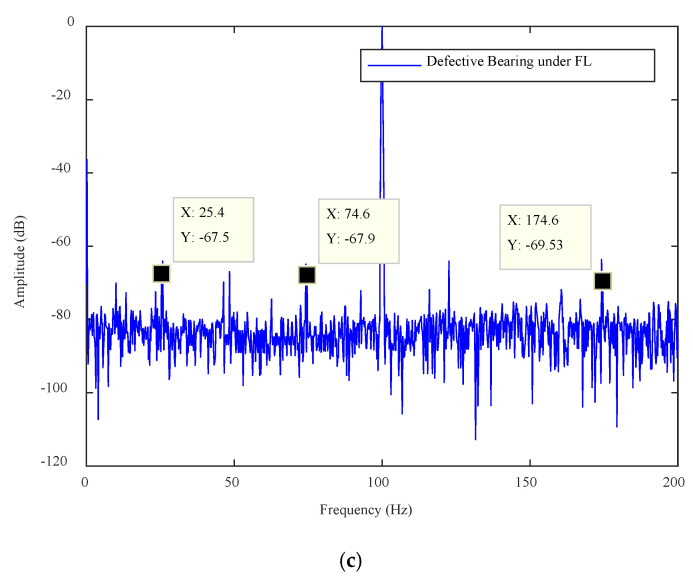
The FL plots for the defective bearing: (**a**) normal bearing, (**b**) type 1 defect and (**c**) type 2 defect.

Plots of the IPA signals for no load (NL), medium load (ML) and full load (FL), for a normal bearing and defective bearing (type 1 and type 2), are shown in Figure 6, Figure 7 and Figure 8. The frequencies are plotted on the X-axes and amplitudes are plotted on the Y-axes. In the graphs, ″X″ denotes the frequency and ″Y″ denotes the amplitude at that frequency. The normal bearing data was taken as the baseline data and any deviation of amplitudes from the baseline data was considered as the presence of a fault. An amplitude difference (AD) of 4 dB was recorded for the type 1 fault and of 8 dB for the type 2 fault under NL operation. Similarly, ADs of 10 dB and 12 dB were found in the case of ML. Finally, ADs of 14 dB and 16 dB were observed in the FL operation of the motor. A summary of the amplitude calculations for various scenarios is given in Table 2.

The amplitude differences (were measured by analyzing the plots of the normal bearing and defective bearing and they are listed in Table 2. The AD values were calculated for each case to confirm the presence of the fault. The case where AD > 0 was considered as a faulty case. The motor operates in the industrial environment and any noise level adversely affects the signal amplitudes. This noise can create a false alarm. Thus, a threshold was established and the amplitudes of the measured signal were compared with the threshold value. Those signal features whose amplitudes were greater than the statistical threshold were considered as strong features and rest of the features were considered as weak features. The algorithm designed to accomplish this process was called the SFSEA.

## 5. Fault Classification Algorithm

The bearing features were extracted using the SFSEA and were fed to a machine learning algorithm for the machine health classification. The extreme gradient boosting (XGB) classifier was used as a machine learning algorithm to classify nine different classes of machine health conditions. The XGB classifier is known for its fast execution speed and high classification performance in several domains [37,38]. Therefore, it was used to classify the machine’s health conditions. The nine classes were NL (no load with no defect), NL1 (no load with type 1 defect), NL2 (no load with type 2 defect), ML (medium load with no defect), ML1 (medium load with type 1 defect), ML2 (medium load with type 2 defect), FL (full load with no defect), FL1 (full load with type 1 defect) and FL2 (full load with type 2 defect). The three features used to classify were Y1 (amplitude in dB against first frequency point, i.e., X1), Y2 (amplitude in dB against second frequency point, i.e., X2) and Y3(amplitude in dB against third frequency point, i.e., X3). All these points (X1, Y1; X2, Y2; X3, Y3) are shown in Figure 6, Figure 7 and Figure 8. These classes and features are shown in Table 3.

To identify the strength of each feature in classifying the machine health, seven experiments were conducted, as shown in Table 4, using different feature combinations. An ✔ indicates when a feature was used to classify the nine machine health conditions and an ✖ indicates when the particular feature was not used for classification. For instance, in E1, only the Y1 feature was used for classification while others were not used. Moreover, in E7, all features were used to classify the machine health states. The dataset contained 640 samples of all classes. For cross-validation, a 70/30 train/test split was used, where 70% of the data samples (448 samples) were used to train the XGB classifier and the remaining 30% (192 samples) were used to test and to compute the performance. The XGB parameters were set as: max_depth = 50, min_child_weight = 1, n_estimators = 100, n_jobs = −1, verbose = 1, learning_rate = 0.1. The experimentation was performed using the XGBboost library for Python [39].

The findings of the proposed XGB classifier using different experiments are presented in Table 4 and Table 5. It is quite evident from the results that almost all combinations achieved performances of above 96% and most of the experiments (five out of seven) achieved performances of 100%. One interesting fact is that the Y2 feature alone was able to classify all the nine classes of machine health conditions with 100% performance, and Y1 and Y3 alone achieved performances of 96.57% and 99.69%, respectively. All the feature remaining combinations achieved performances of 100% regardless of the number of features used. The respective confusion matrices of the first four experiments are presented in Table 5.

These findings are quite encouraging and show the strength of the proposed SFSEA for feature extraction and of the XGB-based machine learning model in classifying the nine different machine health conditions. The analysis further emphasizes the fact that even a single feature (Y2) is sufficient to achieve 100% performance in classifying all the different health conditions.

The performances of the developed SFSEA and XGB algorithm were compared with other published papers. The study [28] was chosen as a benchmark study as the authors used a similar experimental framework, a similar number of samples and similar bearing fault types. The authors of [28] compared the performances of the support vector machine algorithm, a k-nearest neighbor network and a convolutional neural network. They reported classification accuracy of 83.04% for the SVM algorithm, 87.85% for the k-NN network and 89.26% for the CNN. However, the techniques proposed in this paper using SFSEA for feature extraction and XGB as the classifier achieved an accuracy of 100%. Thus, the method proposed in this paper outperformed the SVM, k-NN and CNN algorithms.

## 6. Conclusions

The accurate and cost-effective fault detection of centrifugal water pumps has gained great importance in modern industry and this paper therefore presented an intelligent condition monitoring system for the fault diagnosis of water pump bearings. A mathematical framework to identify the fault harmonics in the IPS data was established, and then a novel SFSEA was developed to extract the features from the IPS spectrum. Three fault features identified in the spectrum were extracted and compared with the baseline data to verify the amplitude difference. The fault feature amplitudes were compared with the threshold to remove the impact of noise. An XGB classifier was developed to classify nine health conditions of the water pump and it was found that the proposed method achieved 100% classification accuracy. The significance of the developed method is evident from the performance comparison with other state-of-the-art techniques, namely the SVM, k-NN and CNN algorithms.

## Figures and Tables

**Table 1:** The location of fault features in the IPS under various shaft loads.

Shaft Load	Shaft Speed (Revolutions per Minute)	The Locations of Fault Features in the Spectrum (Hz)		
		X_1_	X_2_	X_3_
0%	1490	79.4	20.6	179.4
50%	1452	77.4	22.6	177.4
100%	1400	74.6	25.4	174.6

**Table 2:** The identification of strong features and weak features through the SFSEA.

Load	Normal Bearing Amplitude (dB)	Defect Class	Defective Bearing Amplitude (dB)	AD (dB)	Statistical Threshold (dB)	Comments
NL	−81.44 −80.54 −81.22	Type 1	−77.44 −76.39 −77.16	4 4.15 4.06	−73.9 dB	Weak features
		Type 2	−73.55 −71.8 −72.6	7.89 8.7 8.62		Strong features
ML	−84.49	Type 1	−74.37	10.12	−73.9 dB	Weak feature
	−84.77		−74.6	10.17		Weak feature
	−79.42		−69.12	10.40		Strong feature
	−84.49	Type 2	−72.1	12.40	−73.9 dB	Strong feature
	−84.77		−72.95	11.82		Strong feature
	−79.42		−67.72	11.7		Strong feature
FL	−84.55	Type 1	−70.35	14.20	−73.9 dB	Strong feature
	−83.19		−70.09	13.10		Strong feature
	−85.25		−71.07	14.18		Strong feature
	−84.55	Type 2	−67.5	17.05	−73.9 dB	Strong feature
	−83.19		−67.9	15.29		Strong feature
	−85.25		−69.53	15.72		Strong feature

**Table 3:** Description of classes and features.

Classes	Description of Classes	Features	Description of Features
NL	No load with no defect	Y1	Amplitude in db against first frequency point, i.e., X1
		Y2	Amplitude in db against second frequency point, i.e., X2
		Y3	Amplitude in db against third frequency point, i.e., X3
NL1	No load with type 1 defect	Y1	Amplitude in db against first frequency point, i.e., X1
		Y2	Amplitude in db against second frequency point, i.e., X2
		Y3	Amplitude in db against third frequency point, i.e., X3
NL2	No load with type 2 defect	Y1	Amplitude in db against first frequency point, i.e., X1
		Y2	Amplitude in db against second frequency point, i.e., X2
		Y3	Amplitude in db against third frequency point, i.e., X3
ML	Medium load with no defect	Y1	Amplitude in db against first frequency point, i.e., X1
		Y2	Amplitude in db against second frequency point, i.e., X2
		Y3	Amplitude in db against third frequency point, i.e., X3
ML1	Medium load with type 1 defect	Y1	Amplitude in db against first frequency point, i.e., X1
		Y2	Amplitude in db against second frequency point, i.e., X2
		Y3	Amplitude in db against third frequency point, i.e., X3
ML2	Medium load with type 2 defect	Y1	Amplitude in db against first frequency point, i.e., X1
		Y2	Amplitude in db against second frequency point, i.e., X2
		Y3	Amplitude in db against third frequency point, i.e., X3
FL	Full load with no defect	Y1	Amplitude in db against first frequency point, i.e., X1
		Y2	Amplitude in db against second frequency point, i.e., X2
		Y3	Amplitude in db against third frequency point, i.e., X3
FL1	Full load with type 1 defect	Y1	Amplitude in db against first frequency point, i.e., X1
		Y2	Amplitude in db against second frequency point, i.e., X2
		Y3	Amplitude in db against third frequency point, i.e., X3
FL2	Full load with type 2 defect	Y1	Amplitude in db against first frequency point, i.e., X1
		Y2	Amplitude in db against second frequency point, i.e., X2
		Y3	Amplitude in db against third frequency point, i.e., X3

**Table 4:** Summary of the experiments conducted to find the strength of feature combinations in classifying machine health.

Experiment (E)	Features Used			Accuracy, %
	Y1	Y2	Y3	
E1	✔	✖	✖	96.57
E2	✖	✔	✖	100
E3	✖	✖	✔	99.69
E4	✔	✔	✖	100
E5	✔	✖	✔	100
E6	✖	✔	✔	100
E7	✔	✔	✔	100

**Table 5:** Confusion matrices of selected experiments.

**(a) E1**	**(b)E2**
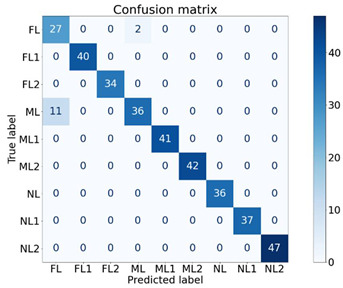	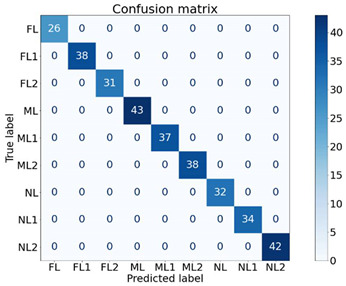
**(c) E3**	**(d) E4**
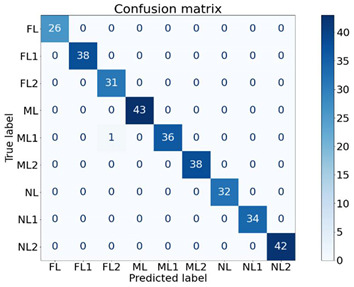	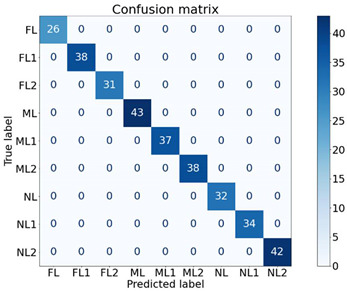

## Data Availability

Not applicable.
